# Intrinsic short-tailed azole resistance in mucormycetes is due to an evolutionary conserved aminoacid substitution of the lanosterol 14α-demethylase

**DOI:** 10.1038/s41598-017-16123-9

**Published:** 2017-11-21

**Authors:** Rita Caramalho, Joel D. A. Tyndall, Brian C. Monk, Thomas Larentis, Cornelia Lass-Flörl, Michaela Lackner

**Affiliations:** 1 Division of Hygiene and Medical Microbiology, Medical University of Innsbruck, Schöpfstraße, number 41, 2nd floor, A-6020 Innsbruck, Austria; 2 School of Pharmacy, University of Otago, Dunedin, 9054 New Zealand; 3 Sir John Walsh Research Institute and the Department of Oral Sciences, New Zealand’s National Centre for Dentistry, University of Otago, Dunedin, 9054 New Zealand

## Abstract

Mucormycoses are emerging and potentially lethal infections. An increase of breakthrough infections has been found in cohorts receiving short-tailed azoles prophylaxis (e.g. voriconazole (VCZ)). Although VCZ is ineffective *in vitro* and *in vivo*, long-tailed triazoles such as posaconazole remain active against mucormycetes. Our goal was to validate the molecular mechanism of resistance to short-tailed triazoles in Mucorales. The paralogous cytochrome P450 genes (CYP51 F1 and CYP51 F5) of *Rhizopus arrhizus*, *Rhizopus microsporus*, and *Mucor circinelloides* were amplified and sequenced. Alignment of the protein sequences of the *R. arrhizus*, *R. microsporus*, and *M. circinelloides* CYP51 F1 and F5 with additional Mucorales species (n = 3) and other fungi (n = 16) confirmed the sequences to be lanosterol 14α-demethylases (LDMs). Sequence alignment identified a pan-Mucorales conservation of a phenylalanine129 substitution in all CYP51 F5s analyzed. A high resolution X-ray crystal structure of *Saccharomyces cerevisiae* LDM in complex with VCZ was used for generating a homology model of *R. arrhizus* CYP51 F5. Structural and functional knowledge of *S. cerevisiae* CYP51 shows that the F129 residue in Mucorales CYP51 F5 is responsible for intrinsic resistance of Mucorales against short-tailed triazoles, with a V to A substitution in Helix I also potentially playing a role.

## Introduction

A small fraction of the more than five million fungal species cause infections in humans. The genetic plasticity of many of these pathogens allows relatively rapid adaption to challenges posed by the host immune system and antifungal therapy^1^, while other pathogens appear to have innate resistance to some classes of antifungals. Most pathogenic fungi are members of the phylum Ascomycota, followed by the Basidiomycota^2^. The ascomycetes contain many common human pathogens from the genera *Aspergillus*, *Candida*, and *Pneumocysti*s^3^, as well as emerging human pathogens including *Fusarium*, *Scedosporium*, *Histoplasma*, and *Coccidioides* ^4^. Among the basidiomycetes, prominent human pathogens are found in the genera *Cryptococcus* ^5^, *Malassezia* ^6^, and *Trichosporum* ^7^. Mucormycetes (previously zygomycetes) include the taxa Mucoromycotina (Mucorales) and Entomophtoromycotina (Entomophtorales). Members of the Mucoromycotina cause systemic human infections. In contrast, members of the Entomophtorales rarely infect humans and are predominantly isolated from cutaneous infections (e.g. *Conidiobolus* spp. and *Basidiobolus* spp.)^8^.

The Mucorales are among the most ancient groups within the fungal kingdom^9^. Like most fungal pathogens, many are ubiquitous saprophytes^9^. They cause highly destructive and potentially lethal infections (mucormycoses) in immunocompromised individuals with specific predisposing conditions (e.g. severe burns, neutropenia, diabetes mellitus)^10^. Worldwide, the most common genera found to cause mucormycosis are *Rhizopus*, followed by *Lichtheimia*, and *Mucor*, with the most frequent clinical isolates being *R. arrhizus* and *R. microsporus* ^8^. Mucormycosis has a high mortality rate, with invasive mucormycosis lethal in up to 96% of cases^11–13^. Although uncommon, mucormycosis is recognized as an emerging disease with limited treatment options. There is also an increasing incidence of breakthrough infections among immunocompromised patients and high-risk patient cohorts receiving voriconazole (VCZ) prophylaxis^14,15^. International guidelines for the treatment of mucormycoses suggest first-line antifungal therapy with liposomal or lipid formulations of amphotericin B (AMB), whereas posaconazole (PCZ) is recommended as salvage therapy^16^. VCZ and fluconazole (FLC) are short-tailed triazoles and, unlike the long-tailed triazole PCZ, have no *in vitro* ^17,18^ or *in vivo* ^19–21^ activity against mucormycetes.

Inhibition of lanosterol 14α-demethylase (LDM) by azole drugs blocks ergosterol biosynthesis and results in a build-up of toxic sterols^22^. Despite extensive knowledge of acquired triazole resistance in ascomycetes (e.g. *Aspergillus* and *Candida species*), the mechanism of innate resistance of mucormycetes to short-tailed triazoles (VCZ and FLC) has yet to be elucidated. Innate FLC resistance (or reduced susceptibility) is frequently found among various human pathogenic fungi (e.g. *Candida krusei*, *Candida glabrata*, *Aspergillus fumigatus*, and Mucorales)^18,23–26^, however innate VCZ resistance is rare. Like *A. fumigatus*, Mucorales appear to possess two *CYP51* paralogues. These are orthologues to *CYP51B* and *CYP51A* and are named *CYP51 F1* and *CYP51 F5*, respectively, according to the Nelson’s database nomenclature^27^. Intrinsic FLC-resistance in *A. fumigatus* has been attributed to a naturally occurring isoleucine 301 (I301) residue in CYP51A^28^.

The lack of *in vitro* activity of FLC and VCZ against Mucorales^17,18,29^, as well as the increased incidence of breakthrough mucormycoses in the last two decades that has coincided with the widespread use of such triazoles in prophylactic and empiric antifungal therapy in high-risk patients^30,31^, suggests that the Mucorales are innately resistant to short-tailed azoles. Furthermore, the cell membranes of mucormycetes contain ergosterol and both mid- (e.g. isavuconazole, IVZ) and long-tailed azoles (e.g. PCZ) usually provide effective treatments. As the Mucorales appear to have a pair of LDMs encoded in separate *CYP51* genes, it seems likely that one or both LDMs might confer resistance to short-tailed azole drugs. Common mechanisms of acquired azole resistance (single-, cross-, or pan-azole) involve non-synonymous single nucleotide point mutations (nsSNPs) that modify the LDM and reduce its affinity for one or more types of triazole drug. On the other hand, the resistance pattern of Mucorales might be considered innate due to one or more ancient amino acid substitutions.

We investigated the molecular basis of innate resistance to short-tailed azoles in Mucorales by: (1) sequencing *CYP51* paralogues in three Mucorales species (*Rhizopus arrhizus*, *Rhizopus microsporus*, and *Mucor circinelloides*), (2) comparing these LDM primary sequences to those of the Mucorales *M. ambiguus*, *Parasitella parasitica*, and *Absidia glauca* and the non-Mucorales species *Saccharomyces cerevisiae*, *Histoplasma capsulatum*, *Coccidioides posadasii*, *Coccidioides immitis*, *Pneumocystic carinii*, *Scedosporium apiospermum*, *Zymoseptoria tritici*, *Uncinula necator*, *A. fumigatus*, *Aspergillus flavus*, *Candida albicans*, *C. glabrata*, *Candida tropicalis*, *Candida dubliniensis*, *Malassezia globosa*, *Cryptococcus neoformans var. grubii* and *Cryptococcus gatti*, and (3) integrating biochemical studies and structural analysis based on a homology model of *R. arrhizus* CYP51 F5 in complex with VCZ, to assess the relevance of nsSNPs to innate azole resistance.

## Results

### Drug susceptibilities

The minimum inhibitory concentrations (MICs) of the Mucorales species *R. arrhizus*, *R. microsporus*, and *M. circinelloides* were determined for the short-tailed triazole drugs VCZ, FLC, and triadimenol (TDM). The MICs for VCZ were as follows: *R. arrhizus* median MIC 16.00 mg/L; *R. microsporus* median MIC 16.00 mg/L; and *M. circinelloides* median MIC 32.00 mg/L (Table 1). The MICs of all tested strains of the three species were >64.00 mg/L for FLC and >16.00 mg/L for TDM (Supplementary Table S1). In contrast, the median MICs of the same strains were 2.00–8.00 mg/L for the long-tailed triazole PCZ and 0.50–2.00 mg/L for the polyene antibiotic amphotericin B, depending on the species tested^32^.

**Table 1:** VCZ susceptibility of Mucorales strains and molecular description of *CYP51* genes and proteins.

species	*Whole cell* susceptibility to VCZ^a^	*CYP51* gene			ORF	LDM		Ref.
	Median (range) MIC (mg/L) (n strains tested)	Designation^b^	*CYP51* family^c^	total length (nt)	total length (nt)	total length (aa)	aa in position 140^d^	
*R. arrhizus*	16.00 (4.00 –>16.00)	Ra*CYP51*A	*F5*	1755	1533	510	F	56
	(n = 17)	Ra*CYP51*B	*F1*	1755	1533	510	Y	
*R. microsporus*	16.00 (4.00–16.00)	*RmCYP51*A	*F1*	1774	1533	510	Y	52
	(n = 13)	*RmCYP51*B	*F5*	1645	1539	508	F	
*M. circinelloides*	16.00 (16.00–>16.00)	*McCYP51*A	*F1*	1773	1539	512	Y	57
	(n = 18)	*McCYP51*B	*F5*	1658	1533	510	F	

^a^VCZ susceptibility according to CLSI standard microbroth dilution method^54^; ORF, open reading frame; LDM, lanosterol 14α-demethylase; MIC, minimum inhibitory concentration; ^b^loci A and B were attributed according to identification with *CYP51A* and *CYP51B* of *A. fumigatus* strain AF293; P450 family name was attributed to each *CYP51* paralogue according to Nelson’s database (http://drnelson.utmem.edu/CytochromeP450.html); ^d^amino acid position 140 according to *S. cerevisiae CYP51* numbering; nt, nucleotide; aa, amino acid; Ref, reference. Additional information on *in vitro* PCZ and amphotericin B susceptibilities can be found in a previous study^32^.

### DNA sequence analysis of Mucorales CYP51s

The paralogous pairs of *CYP* genes in *R. arrhizus*, *R. microsporus*, and *M. circinelloides* were amplified from genomic DNA, sequenced, and their primary amino acid sequences (introns excluded) translated. The lengths of the nucleic acid and primary sequences of *CYP51 F5* and *CYP51 F1* are shown in Table 1. Each open reading frame (ORF) was interspersed with at least 2 introns (2 introns for *R. arrhizus* and *M. circinelloides CYP51 F5*s, 3 in *R. microsporus CYP51 F5*s, and 4 in all *CYP51 F1*s). The protein sequences were of 508–510 amino acids for CYP51 F5 and 510–512 amino acids for CYP51 F1.

### Alignment of CYP51 F5 and CYP51 F1 primary sequences

**Figure 1:**
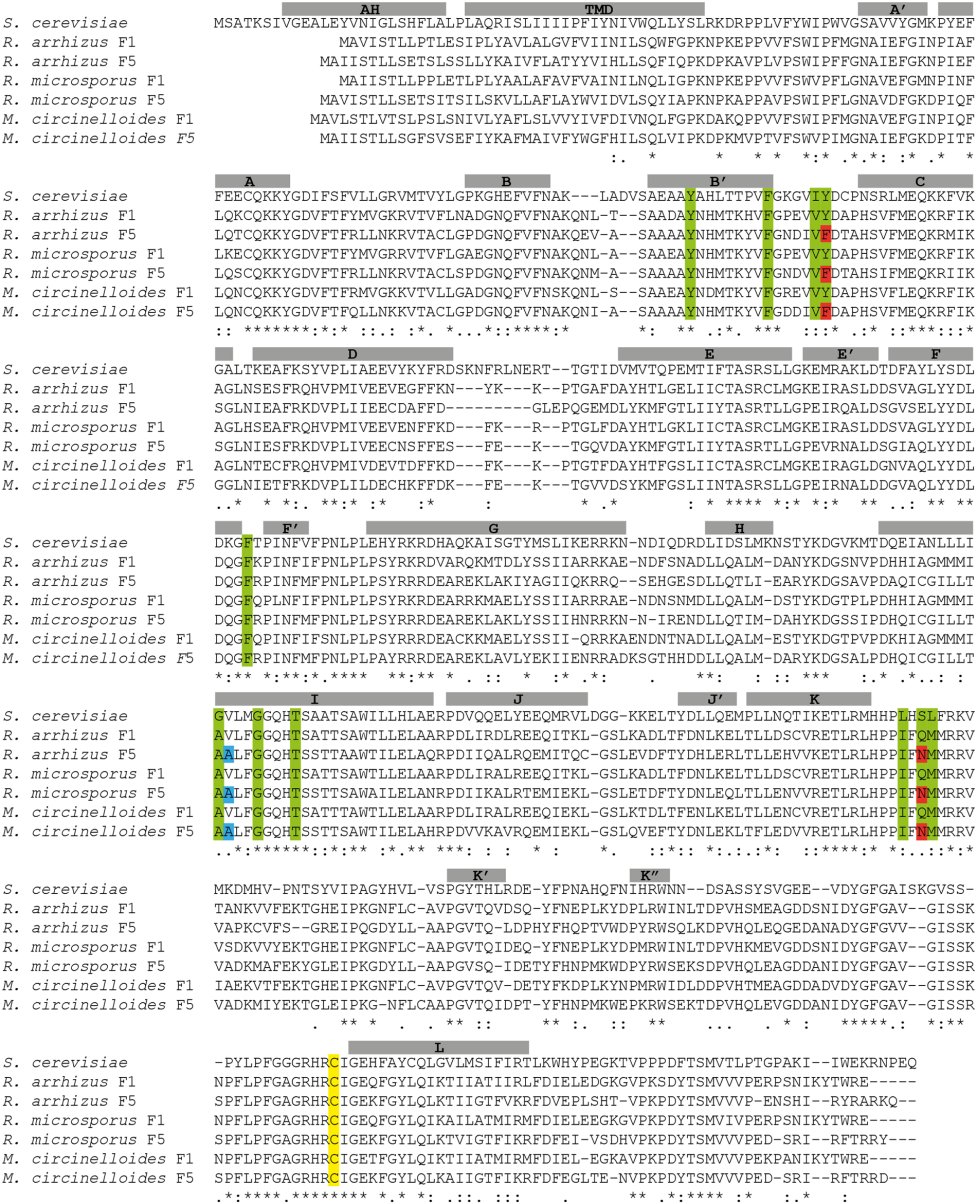
Sequence alignment of *Saccharomyces cerevisiae* lanosterol 14-α demethylase with six Mucorales CYP51s. *R. arrhizus F1* (n = 17) and *F5 CYP51s* (n = 3), *R. microsporus F1* and *F5 CYP51s* (n = 13 for both LDMs) and *M. circinelloides F1* and *F5 CYP51s* (n = 18 for both LDMs) were aligned against *S. cerevisiae CYP51* using T-coffee (Expresso)^61^. Residues within 4 Å of voriconazole in the structure *S. cerevisiae CYP51* (PDBID: 5HS1^43^) are highlighted in green. Those highlighted in red correspond to resistance mutations in *CYP51 F5*. The V to A substitution is shown in blue. Helices from the voriconazole *CYP51* complex are shown above the alignment in gray and the heme coordinating cysteine is shown in yellow. The final two residues of the *S. cerevisiae* sequence are not shown. MH, Membrane-associated helix; TMD, transmembrane domain.

The CYP51 F1 and F5 primary sequences from the three Mucorales species showed >98.0% sequence identity. The *R. arrhizus* CYP51 F5s (3 strains) showed 100% identity and their CYP51 F1s (17 strains) were 99.4% identical (4 amino acid substitutions [aas]). The *R. microsporus* CYP51 F5s (13 strains sequenced for both genes) had 98.4% sequence identity (8 aas), whereas their CYP51 F1s showed 99.6% sequence identity (3 aas). *M. circinelloides* CYP51 F5s (18 strains sequenced for both genes) exhibited 99.9% sequence identity (1 aas) and their CYP51 F1s showed 100.0% sequence identity. Alignment of the consensus sequence of the *R. arrhizus*, *R. microsporus*, and *M. circinelloides* CYP51 F5s with the consensus sequence for their CYP51 F1s gave an overall pairwise sequence identity of 64.9% (Fig. 1, percentage identities calculated using the pairwise alignment tool from Geneious™ software v. 8.1.9, Biomatters Limited, Auckland, NZ).

**Figure 2:**
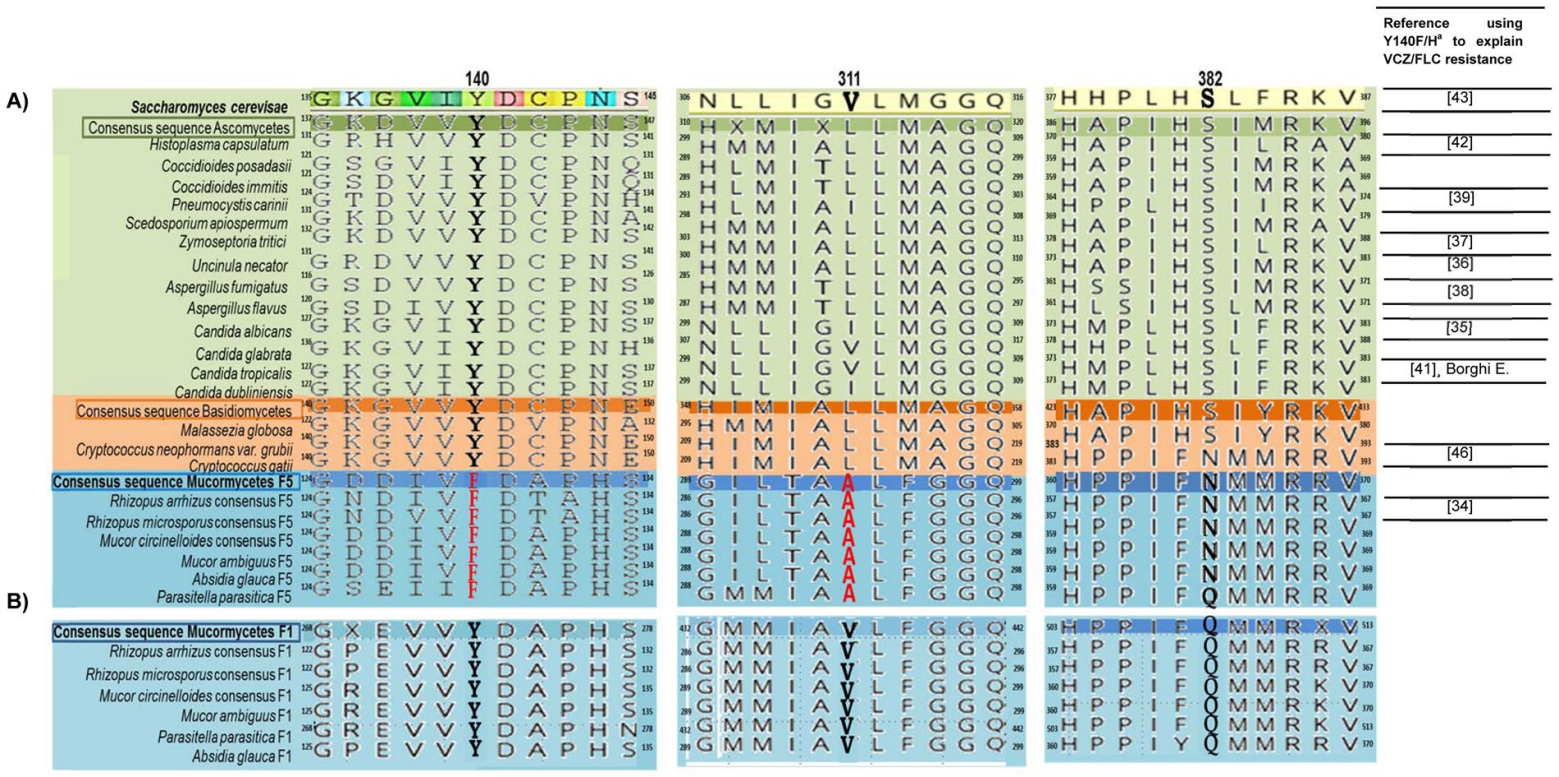
Amino acid sequence alignment of lanosterol 14-α demethylase in human pathogenic fungi. Proteins belonging to sixteen fungi with consensus sequences of six Mucorales *CYP51s*. (**A**) Alignment using *Saccharomyces cerevisiae* Erg11 as reference (partial amino acid sequence colored) compared with wild-type ascomycete strains (n = 13) (in light green) with phylum consensus (dark green); wild-type basidiomycetes (n = 3) (light orange) with phylum consensus sequence (dark orange); sequences of *R. arrhizus* (n = 3), *R. microsporus* (n = 13), and *M. circinelloides* (n = 18) *CYP51 F5* sequences, together with *Mucor ambiguus*, *Parasitella parasitica*, and *Absidia glauca* LDM sequences (light blue), with subphylum consensus sequence (dark blue). The frequently mutated tyrosine residue homologous to *S. cerevisiae* CYP51 Y140 is marked in bold, and the change to phenylalanine is seen in all our CYP51 F5 Mucorales species (original position F129) shown in red. The V to A substitution in position 311 (*S. cerevisiae* numbering) is only observed among Mucorales LDM F5 sequences, and are depicted in red. The Q/N side chains aligning with *S. cerevisiae* LDM S382 are marked in bold. Superscript numbers show the positions of the 5′ and 3′ amino acids for each sequence. The last column gives references reporting a mutation homologous to Y140F/H as responsible for short-tailed azole resistance in the species indicated; ^a^according to *S. cerevisiae* numbering. (**B**) Shows the same alignment with consensus sequences of six Mucorales CYP51 F1s. The residue homologous to ScErg11p Y140 is marked in bold and is seen also in the protein sequence of the six Mucorales CYP51 F1s. In position 311 (*S. cerevisiae* numbering), a V is also present in all Mucorales CYP51 F1s. The Q/N side chains are also present in Mucorales CYP51 *F1*, marked in bold, and in both LDMs do not affect VCZ binding.

To enlarge the Mucoromycotina data set and for comparison with the ascomycetes and basidiomycetes as representatives of the wider fungal kingdom, we aligned the Mucorales CYP51 F1 and F5 consensus protein sequences (Fig. 2A and B for partial CYP51 F5 and CYP51 F1 alignments, respectively) against CYP51 sequences of *Mucor ambiguus*, *Parasitella parasitica*, *Absidia glauca*, and 16 other fungal pathogens as well as *Saccharomyces cerevisiae* (Supplementary Table S2). The alignment between all 6 Mucorales CYP51 F5s gave a sequence identity of 75.8%, and the 6 CYP51 F1s gave a pairwise sequence identity of 81.2%. Primary sequence alignments with the wider fungal data set showed pairwise sequence identity of 46.7% with the consensus sequence for our three Mucorales CYP51 F5s and 48.0% sequence identity with the consensus sequence for the three Mucorales CYP51 F1s (data not shown). The high (>46.0%) sequence identities with the wider data set, including the presence of conserved signatures of the P450 proteins, such as the EXXR (ETLR Mucorales sequence) motif in the K-helix and the CXG in the heme-binding domain^33^ in both Mucorales proteins, strongly indicatesthat these CYPs are indeed LDMs (Figs 1, and S1).

Real-time PCR (qPCR) found that both *CYP51* genes are expressed in all mucormycetes strains tested. Two qPCR experiments specifically targeting the *CYP51 F1* and *F5* transcripts of *R. arrhizus*, *R. microsporus*, and *M. circinelloides* were performed as described in Methods using specific primers (Supplementary Table S3). Both genes were expressed with or without exposure for 48 h to either FLC or itraconazole (ITC) at 0.5 mg/L. As an example, the PCR product obtained for *CYP51 F5* cDNA is illustrated in Supplementary Fig. S2A. DNA sequence analysis of the amplicons obtained for both *CYP51 F1* and *F5* RNAs confirmed their identity (Supplementary Fig. S2B).

Consensus primary sequences for CYP51 F1 and F5 were aligned against each other, the CYP51s of other fungal pathogens and *S. cerevisiae* (Figs 1 and 2) to screen for amino acid substitutions previously identified as conferring resistance to short-tailed azoles in different fungal species^34–42^. For example, *S. cerevisiae* LDM and CYP51 F1 retained a tyrosine residue that aligned to tyrosine 140 (Y140, *S. cerevisiae* numbering i.e. Y129 in *M. circinelloides*, Y129 in *R. arrhizus* and *R. microsporus*). In contrast, the alignment identified a phenylalanine 129 substitution (129F, Y140F *S. cerevisiae* numbering) conserved in all Mucorales CYP51 F5s. The presence of F129 in the CYP51 F5 of all 6 Mucorales species, including *R. arrhizus*, *R. microsporus*, and *M. circinelloides*, is consistent with a unique naturally occurring amino acid substitution in Mucorales CYP51 F5.

Until recently, the effects of substitutions equivalent to Mucorales CYP51 F5 Y129F had not been assessed at the structural level. Sagatova *et al*. used a full-length hexahistidine-tagged *S. cerevisiae* CYP51 to determine phenotypes and obtain high resolution X-ray crystal structures of wild type and Y140F/H mutant enzymes complexed with short-tailed (VCZ, FLC) and long-tailed (PCZ, ITC) triazoles^43^. The Y140F/H mutations disrupted a hydrogen bond network in the active site involved in the binding of short-tailed azoles, such as FLC and VCZ and lead to resistance.

**Figure 3:**
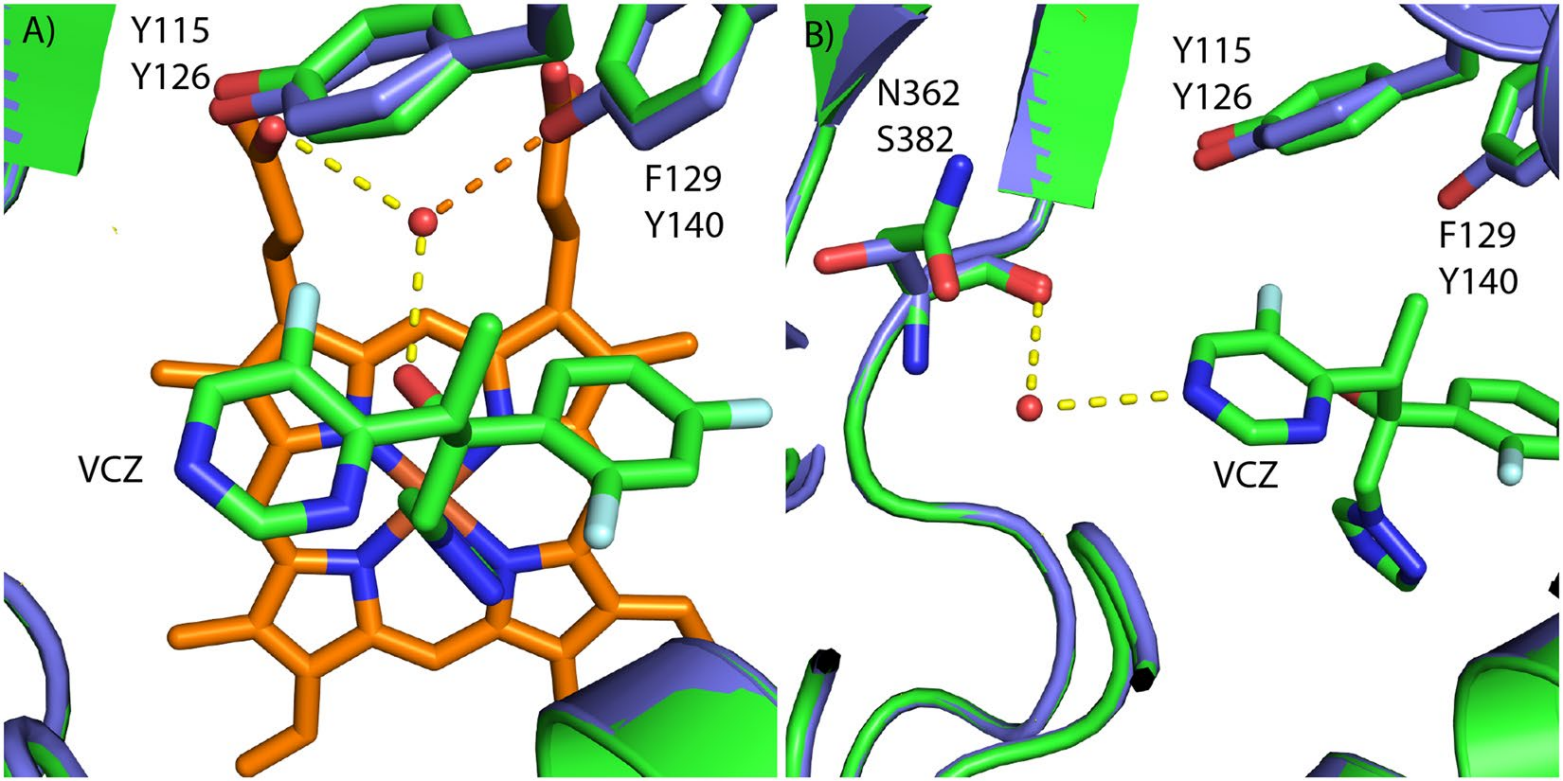
Homology model of *R. arrhizus* LDM F5. (**A**) Superimposition showing the active site of *R. arrhizus* LDM F5 (green) showing the position of F129 in relation to *S. cerevisiae* Y140 (purple, PDB ID: 5HS1)^43^. Voriconazole (green carbons, blue nitrogens, red oxygens and cyan fluorines) is shown coordinating to the heme (orange) in the homology model. The key water-mediated hydrogen bonding network centered on the water molecule (red sphere) with hydrogen bonds is shown by dashed lines. The orange dashed line shows the hydrogen bond that is lost in the presence of phenylalanine (F129) substitution compared with tyrosine (Y140). The water molecule is present only in the crystal structure. (**B**) Substitution of serine (*S. cerevisiae* S382) for asparagine (*R. arrhizus* LDM F5 N362) can be seen adjacent to VCZ and shows no polar interactions. This is highlighted by the superimposition of the homology model (green) onto the crystal structure (purple). The water molecule is present only in the crystal structure.

We used the 2.1 Å resolution X-ray crystal structure of *S. cerevisiae* LDM in complex with VCZ (PDB ID: 5HS1)^43^, as the template for a homology model of the Mucorales CYP51 F5 protein. From the initial alignment, eleven Mucorales CYP51 amino acids that line the active site cavity within 4 Å of the VCZ are highlighted in green (Fig. 1). Six of these residues are identical to those found in wild type *S. cerevisiae* LDM and the remaining five involve conservative amino acid substitutions. Only two of the latter substitutions differ between the Mucorales F1 and F5 CYP51s and potentially contribute to azole resistance. The amino acid substitution homologous to Y140F in the ScCYP51 primary sequence has been characterized at the structural (PDB ID: 4ZE0, 2.2 Å resolution) and functional levels, and confers significant resistance to the short-tailed triazoles FLC and VCZ, but not the long-tailed triazoles ITC and PCZ^43^. The *S. cerevisiae* LDM Y140F structure shows the loss of a water-mediated hydrogen bonding network between the hydroxyl of Y140, the tertiary alcohol of VCZ (or FLC) and a heme propionate via a highly conserved water molecule. The loss of this hydrogen bond has no impact on the binding of ITC (PDB ID: 5EQB) or PCZ (PDB ID; 4ZE1) because these drugs are able to fill the space occupied by the key water^43,44^. The *R. arrhizus* CYP51 F5 homology model clearly shows F129 corresponds structurally to Y140 in the *S. cerevisiae* LDM structure (Fig. 3A).

Another amino acid substitution, from S382 in wild type *S. cerevisiae* LDM to the amide side chain of the Mucorales LDM F5 N362, occurs adjacent to the pyrimidine ring of VCZ. We deduce that the Q/N side chains that structurally align with *S. cerevisiae* LDM S382, do not affect VCZ binding/susceptibility, despite the F129 substitution in the LDM of Mucorales species always being matched with a N362 substitution (Figs 2A and 3B). In addition, the crystal structure of *S. cerevisiae* LDM Y140F in complex with PCZ (PDB ID: 4ZE1) shows a key water that forms hydrogen bonds with the piperazine ring of the drug as well as to the main chain carbonyl and oxygen of S382. These findings are consistent with the Q/N362 substitutions in the Mucorales CYP51s not having a significant impact on the binding of either PCZ or VCZ (Fig. 1). Analysis of the immediate environment of V311 in *S. cerevisiae* LDM, which corresponds to the V to A substitution in Mucorales LDM F5, shows that it is close to the heme but does not directly interact with VCZ despite its main chain being within 4 Å of the drug. The substitution may change the geometry of the active site or possibly affect the position of Helix I or heme but only slightly.

Substitutions equivalent to the *S. cerevisiae* LDM Y140F substitution are exclusively found in Mucorales LDM F5 and only seen in mutant strains of other non-Mucorales fungal pathogens. All wild type isolates contained the same residue as Mucorales CYP51 F1 (Y140 in *S. cerevisiae*) (Fig. 2B).

## Discussion

Several fungal pathogens of humans are innately resistant to one or more of the azole antifungal drugs. Fungal pathogens such as *C. krusei* ^23^ and *A. fumigatus* ^23,25,45^ are innately resistant to FLC. Moreover, the heteroresistance seen in *C. neoformans* to FLC has been defined as an innate trait^46^. The innate resistance of *A. fumigatus* to FLC has been proposed to be due to the single amino acid substitution T310I in CYP51A^28,47^, equivalent to T322I in *S. cerevisiae* LDM or T315 in *C. albicans* LDM. *Pneumocystis carinii* is also innately resistant to treatment with azoles^39^. Its LDM has two point mutations (D113E and K125T) comparable to those in some *C. albicans* strains showing reduced susceptibility to FLC and VCZ, but not ITC^39^.

In agreement with previous studies that categorized the Mucorales as innately resistant to VCZ^17,18,29,48–50^, we found high *in vitro* MIC values for VCZ (median MICs: 16.00 mg/L for *Rhizopus* spp. and 32.00 mg/L for *M. circinelloides*). Although high values were consistently observed, relatively variable MICs were found in *R. arrhizus* and *R. microsporus* (e.g. one strain from both species exhibited MICs of 4.00 mg/L). All strains shared the same amino acid substitution in their LDM F5 sequences despite this slight *in vitro* phenotypic variability (within tolerated CLSI testing variability of 2 dilution steps) (Fig. S3). *In vivo Drosophila melanogaster* and mouse models of Mucorales infection showed that exposure to VCZ selectively enhanced virulence and significantly reduced survival rates of the model organisms, when compared to infection with a *R. arrhizus* strain not pre-exposed to VCZ^51^. Patients with pulmonary or splenic mucormycosis fail to respond to VCZ therapy^52^. All Mucorales species tested also showed *in vitro* resistance to the short-tailed triazoles TDM and FLC (median MICs: >16.00 mg/L and >64.00 mg/L, respectively), but variable susceptibility to the long-tailed triazole PCZ (2.00–8.00 mg/L) and the polyene amphotericin B (0.50–2.00 mg/L), demonstrating that Mucorales are resistant to short-tailed triazoles only. This observation accords with previous studies that demonstrated the triazoles VCZ and FLC have poor or no activity against Mucorales^18,26^. This emphasizes the need to communicate why PCZ, but not VCZ or FLC, should be recommended for therapy against Mucorales^20^ and why the long-tailed triazole shows species-dependent antifungal activity^32^. The triazole antifungals that target LDM differ both in the size of the tail and the presence of a tertiary alcohol found in VCZ and FLC, but not present in PCZ. Furthermore, members of the order Mucorales are not only innately resistant to both VCZ and FLC, but can also develop acquired resistance to PCZ^32^. Amino acid substitutions in Mucorales LDMs that differentially affect the binding of short- and long-tailed may explain these phenotypes.

Alignment of the CYP51 primary sequences with the set of fungal pathogen LDMs demonstrate the existence of two Mucorales CYP51s, denoted CYP51 F5 and CYP51 F1, both of which appear to be expressed in the presence of azole drugs. Moreover, the sequence identity between the consensus primary sequences of Mucorales LDMs, 16 other pathogenic fungi and *S. cerevisiae*, are sufficiently high (46.7% for CYP51 F5 and 48.0% for CYP51 F1) to confirm both proteins as members of the CYP51 family of cytochrome P450 (i.e. sterol demethylases)^53^. The alignment also showed that the polar, uncharged aromatic residue Y127 or Y129 in Mucorales CYP51 F1 (Y140 in *S. cerevisiae* numbering), is not conserved in Mucorales CYP51 F5 and is naturally substituted by the non-polar, aromatic F129 (Y140 in *S. cerevisiae* numbering) residue.

Previous work on the molecular basis for azole susceptibility in *R. arrhizus* identified the equivalent CYP51A (CYP51 F5) and CYP51B (CYP51 F1) sequences including the naturally occurring CYP51A Y129F substitution^34^. Furthermore, expression of *R. arrhizus* CYP51 F5 in yeast gave a >32-fold decrease in susceptibility to VCZ (compared to the same strain expressing the *S. cerevisiae CYP51* gene) while expression of CYP51 F1 did not increase susceptibility. The same group has suggested that the equivalent Y132F (Y140F *S. cerevisiae* numbering) substitution in combination with a T471I substitution in the *C. albicans* LDM significantly reduced susceptibility to VCZ, but not PCZ^35^. Similarly, a *Histoplasma capsulatum* strain harboring the LDM amino acid change Y136F (analogous to Y129F in Mucorales CYP51 F5) showed reduced susceptibility to FLC and VCZ, but not PCZ^42^. While azole resistance in *C. tropicalis* has been ascribed to various missense point mutations in LDM, the single mutation Y132F has a major role in azole resistance^41^. The Y132F substitution has been described as the only mechanism conferring resistance to FLC and VCZ in 10 *C. tropicalis* clinical isolates (Borghi E., Personal Communication, ECCMID 2012). The innate resistance of *C. neoformans* to both FLC and VCZ was explained by a LDM Y145F mutation (analogous to Y140F in *S. cerevisiae*)^40^. *Aspergillus fumigatus* LDM can harbor numerous amino acid changes that contribute to multi-azole resistance, including the single Y121F (analogous to Y140F in *S. cerevisiae*) mutation that appears responsible for reduced susceptibility to VCZ, but not PCZ or ITC^38^. Azole resistant fungal infections have been associated with the widespread use of these agrochemical fungicides since the 1970s. The important fungal phytopathogens *Z. tritici* and *U. necator* respectively, show Y137F and Y136F mutations in LDM (both analogous to Y140F in *S. cerevisiae*) confer reduced susceptibility to the short-tailed triazole TDM, one of the first fungicides used in crop protection^36,37^. The LDMs of these two phytopathogens were also included in our study. The poor *in vitro* susceptibility of Mucorales to TDM supports the principle that this innate resistance applies to all short-tailed azoles that contain a secondary or tertiary alcohol. Furthermore, the susceptibility of Mucorales to drugs like isavuconazole, which also contains a tertiary alcohol, may be due to the additional affinity afforded by its medium-sized tail.

By overexpressing wild type and mutant *S. cerevisiae* LDMs in yeast, determining the phenotypes of the recombinant yeast in response to triazoles and obtaining high resolution X-ray crystal structures, Sagatova *et al*.^43^ elucidated the mechanism responsible for resistance to short-tailed over long-tailed triazoles by showing that the Y140F/H mutation in LDM significantly perturbs a hydrogen bonding network. This information enabled us to use structural alignments to demonstrate that the F129 residue in Mucorales CYP51 F5 is likely to be responsible for reduced susceptibility to short-tailed (VCZ, FLC), but not long-tailed triazoles (PCZ). This idea, including the possible contribution by the V291A (*R. arrhizus* and *R. microsporus*) or V293A (*M. circinelloides*) substitution, can now be experimentally tested by expressing codon optimized versions of recombinant Mucorales CYP51 F1 and F5 enzymes in a suitable yeast host, determining the expression levels of functional enzymes, measuring susceptibilities to azole drugs and obtaining high resolution X-ray crystal structures of the recombinant enzymes in complex with VCZ, IVZ, and PCZ.

In summary, application of the functional analysis and structure-based findings of Sagatova *et al*.^43^ for *S. cerevisiae* CYP51, shows that innate resistance to short-tailed but not long-tailed azoles in at least six Mucorales species appears to be mediated by the substitution Y129F in the loop between helices B’ and C’ together with a V to A substitution in Helix I of the active site of LDM F5, but not LDM F1. As both LDM isoforms are expressed in the presence or absence of VCZ and *R. arrhizus* LDM F5 confers selective resistance when expressed in yeast, we propose that a Y129F substitution of LDM F5 is primarily responsible for the innate resistance to short-tailed azoles observed in Mucorales. This knowledge adds to our understanding of how azoles interact with Mucorales CYP51 F5, and explains why PCZ, but not short-tailed azoles are an effective therapy against mucormycosis. Moreover, our structural models can serve as template to screen *in silico* for novel azole drugs for their activity against mucormycetes.

## Methods

### Strain set identification and susceptibility testing

Forty-eight strains belonging to the species *Rhizopus arrhizus* (n = 17), *R. microsporus* (n = 13), and *Mucor circinelloides* (n = 18) were analyzed. DNA was extracted and strains identified in a previous study^32^.

*In vitro* antifungal susceptibility to VCZ, FLC and TDM (Sigma-Aldrich, St. Louis, MO, USA) was tested (three biological replicates) using the CLSI broth microdilution method (M28-A2)^54^ in a range of 0.03 mg/L – 16.00 mg/L (VCZ and TDM), and 0.13 mg/L–64.00 mg/L (FLC). Minimum inhibitory concentrations (MICs) were read visually after 48 h at 37 °C, with the exception that MICs for *M. circinelloides* were read after 48 h 30 °C^54^. Control strains used were *A. fumigatus* ATCC^®^ 204306, *A. flavus* ATCC^®^ 204304, *C. parapsilosis* ATCC^®^ 22019, and *C. krusei* ATCC^®^ 6258^55^.

### PCR amplification and sequence analysis of the CYP51 families F5 and F1

Whole genome sequence assemblies of *R. arrhizus* ^56^ (GenBank No. KK998513.1), *R. microsporus* ^52^ (GenBank No. CCYT01000001) and *M. circinelloides f. circinelloides* 1006PhL^57^ (GenBank No. AOCY00000000.1) are available online (https://www.ncbi.nlm.nih.gov/genbank/). Gene sequences of the CYP51 homologues used were: *R. arrhizus* (RO3G_11790.3; RO3G_16595.3), *R. microsporus* (RMCBS344292_18743; RMCBS344292_12255), and *M. circinelloides* (HMPREF1544_03888.1; HMPREF1544_08704.1). The paralogues of *CYP51* were named *CYP51 F5* or *CYP51 F1* according to the Nelson’s database nomenclature from the Cytochrome P450 Homepage (http://drnelson.utmem.edu/CytochromeP450.html)^27^. Species-specific primer sequences are presented in Supplementary Table S4. The PCR master mix contained (total volume 20 µl): 10.0 µL 2× KAPA 2 G ROBUST READY MIX^TM^ (KAPA Biosystems Inc., Wilmington, MA USA) (final concentration 0.1 mM MgCl_2_), 1.0 µL of each species-specific primer (final concentration of 0.02 µM each), 2.0 µL of template DNA (final concentration: 1.0–2.5 ng/µL) and 6.0 µL of nuclease-free water. PCR conditions for gene amplification were: initial 95 °C for 3 min, 35 cycles: 95 °C for 15 s, annealing temperature for 15 s (Table 1), and 72 °C for 1 min, final elongation step at 72 °C for 2 min. Amplicons were separated in 1.0% (w/v) agarose gels stained with 2.5 µL GelRed™ dye (Biotium Inc., Hayward, CA, USA). For DNA sequence analysis, amplicons were purified using ExoSAP-IT (USB Corporation, Affymetrix, Santa Clara, CA, USA), according to the manufacturer’s instructions. The sequencing reactions were performed using the BigDye™ Terminator v3.1 Cycle Sequencing Kit (Applied Biosystems, Carlsbad, CA, USA), according to the manufacturer’s instructions and the primers used are given in Supplementary Table S4. The sequencing reaction conditions were as follows: initial denaturation at 96 °C for 1 min, followed by 45 cycles with 96 °C for 10 s, 50 °C for 20 s, 60 °C for 6 min and a final elongation step at 60 °C for 15 min. The PCR products were purified using BigDye XTerminator™ kit (Applied Biosystems, Carlsbad, CA, USA) and the sequence analysis performed on an ABI 3500 Genetic Analyzer (Applied Biosystems, Carlsbad, CA, USA).

### Sequence alignment of the CYP51 Mucorales LDMs against important pathogenic fungi

Amino acid sequences were translated by using Geneious ™ software v. 8.1.9, (Biomatters Limited, Auckland, NZ) and by applying the genetic code 4: molds, protozoans, mitochondrial. Alignment of all translated sequences was performed by using the BLOSUM62 matrix. The CYP51 F5 and F1 primary amino acid sequences of *R. arrhizus*, *R. microsporus*, and *M. circinelloides* were aligned with other mucormycetes (n = 3; *Mucor ambiguus*, *Parasitella parasitica*, and *Absidia glauca*), ascomycetes (n = 13; *Histoplasma capsulatum*, *Coccidioides posadasii*, *Coccidioides immitis*, *Pneumocystis carinii*; *Scedosporium apiospermum*, *Zymoseptoria tritici*, *Uncinula necator*, *Aspergillus fumigatus*, *Aspergillus flavus*, *Candida albicans*, *Candida glabrata*, *Candida tropicalis*, and *Candida dubliniensis*), and basidiomycetes (n = 3; *Malassezia globosa*, *Cryptococcus neoformans var. grubii*, and *Cryptococcus gattii*) using Geneious™ software v. 8.1.9 (Biomatters Limited, Auckland, NZ) with *Saccharomyces cerevisiae* LDM as reference. The relevant accession numbers are given in Supplementary Table S2.

Overall sequence identities for both nucleotide and amino acid sequences were calculated using the pairwise alignment tool from Geneious™ software v. 8.1.9, (Biomatters Limited, Auckland, NZ) and depicted as percentages. The average percent identity over the alignment is given. This was computed by comparing all pairs of bases or amino acids residues in the same column and scoring a hit (one), when they are identical, divided by the total number of pairs.

### Confirmation of the expression of the CYP51 F5 and CYP51 F1 genes

One strain per species was studied for *CYP51* gene expression. *R. arrhizus* CBS 120808, *R. microsporus* F2 and *M. circinelloides* 39.10 were tested by growth in RPMI medium 1640 (Sigma-Aldrich, St. Louis, MO, USA) in the presence or absence of 0.50 mg/L ITC or FLC. Each strain was grown for 4 to 5 days in supplemented minimal medium (SUP)^58^ at 37 °C or 30 °C (for *M. circinelloides*). A final inoculum of 10^6^ spores/mL was cultured in Erlenmeyer flasks containing 100 mL RPMI 1640 liquid medium. Each culture was then shaken for 48 h in the dark at 37 °C or 30 °C, depending on the species tested. The mycelia were harvested using a nylon mesh filter and transferred immediately to liquid nitrogen.

RNA was isolated using the TRI^®^ reagent (Sigma-Aldrich, St. Louis, MO, USA)^59^ and diluted to a final concentration of 2.0 µg/µL. First-strand cDNA synthesis was obtained using the High-Capacity cDNA Reverse Transcription Kit (Thermo Fisher Scientific Inc., Massachusetts, USA). The reaction master mix (final volume 20 µl) consisted of 2.0 µL of 10x Reverse Transcriptase Buffer, 0.8 µL of 25x dNTP Mix (final concentration 4 mM), 2.0 µL 10x Reverse Transcriptase Random Primers, 1.0 µL of Reverse Transcriptase (final concentration 2.5 U/µL), 4.2 µL of nuclease free water and 10.0 µL of RNA (2.0 µg/mL). The reaction was run on a peqSTAR 2x Gradient Thermo-cycler (PeqLab, LLC, GmB) using the program: 25 °C for 10 min, 37 °C for 120 min and 85 °C for 5 min. A 1:100 dilution of cDNA preparations were used for RT-qPCR amplification of *CYP51 F1* and *F5* sequences.

### qPCR design for CYP51 F5 and CYP51 F1

qPCR (Supplementary Table S3) was used to specifically amplify *CYP51 F5* or *CYP51 F1* and confirm their expression. Species-specific primers were design using Geneious™ software v. 8.1.9 (Biomatters Limited, Auckland, NZ). PCR reaction mixtures (final volume: 16 µL) contained 10.0 µL of SsoFast^™^ EvaGreen^®^ Supermix (Bio-Rad Laboratories Incorporated, Hercules, CA, USA), 1.0 µL containing 0.5 µM of each primer, 2.0 µL of ultrapure water and 3.0 µL of each cDNA sample at a final concentration of 2.0 ng/µL. All samples were run in duplicate in three biological replicates. PCR reactions were conducted using a CFX96^™^ real-time PCR detection system (Bio-Rad Laboratories Incorporated, Hercules, CA, USA) using the program: 95.0 °C for 5 min, followed by 40 cycles: 95.0 °C for 15 s, 59.5 °C for 60 s (*CYP51 F5*) or 56 °C for 60 s (*CYP51 F1*) and 72 °C for 60 s. The melting curve was generated using 0.5 °C temperature incensement steps from 60 °C to 95 °C for 5 s. An actin gene fragment was used as housekeeping gene for all gene expression analyses. The PCR mixture and conditions were the same as those described for the amplification of the Mucorales *CYP51 F5* and *CYP51 F1* except the annealing temperature was set at 53.0 °C. The amplicons generated from the *CYP51 F5* and *CYP51 F1* qPCRs were separated in a 2.0% v/w agarose gel to confirm the presence of single products of the desired size (Supplementary Fig. S2A). DNA sequence analysis of the products obtained for both genes confirmed the amplification of the targeted genes and DNA regions (Supplementary Fig. S2B). The sequences were manually checked for the presence of the specific triplets TTY in the *CYP51 F5* genes and the TAY in the *CYP51 F1* genes.

### Homology modeling

The homology model of *R. arrhizus* LDM F5 was developed using modeller v9.15 (http://salilab.org/modeller/modeller.html)^60^ based on the sequence alignment generated from T-coffee (Expresso) (Fig. 1)^61^. A total of 20 models were built using the loop model protocol in modeller with *S. cerevisiae* LDM complexed with VCZ as a template (PDB ID: 5HS1). All models were generated with VCZ bound to the heme group in the active site. The model with the lowest molpdf score was selected for further analysis. Visual analysis was carried out using PyMOL software (Schrödinger Inc., NY, USA).

### Data Availability

Majority of the data generated and analyzed in this study are included in this published article and its Supplementary Material files. Additional supporting datasets, homology model of R. arrhizus LDM F5, and raw data generated during the current study may be provided by the corresponding author on request.

## Electronic supplementary material

Supplementary Information

## References

[1] Perez-Nadales E (2014). Fungal model systems and the elucidation of pathogenicity determinants. Fungal Genet Biol..

[2] De Hoog, G. S., Guarro, J., Gené, J. & Figueiras, M. J. *Atlas of clinical fungi*, *second edition*. 125–225 (American Society for Microbiology, 2000).

[3] Walzer PD (1999). Immunological features of *Pneumocystis carinii* infection in humans. Clin Diagn Lab Immunol..

[4] Tortorano AM (2014). ESCMID and ECMM joint guidelines on diagnosis and management of hyalohyphomycosis: *Fusarium* spp., *Scedosporium* spp. and others. Clin Microbiol Infect..

[5] Shirley RM, Baddley JW (2009). Cryptococcal lung disease. Curr Opin Pulm Med..

[6] Velegraki A, Cafarchia C, Gaitanis G, Iatta R, Boekhout T (2015). *Malassezia* infections in humans and animals: pathophysiology, detection, and treatment. PLoS Pathog..

[7] Walling DM, McGraw DJ, Merz WG, Karp JE, Hutchins GM (1987). Disseminated infection with *Trichosporon beigelii*. Rev Infect Dis..

[8] Ribes JA, Vanover-Sams CL, Baker DJ (2000). Zygomycetes in human disease. Clin Microbiol Rev..

[9] Hoffmann K (2013). The family structure of the Mucorales: a synoptic revision based on comprehensive multigene-genealogies. Persoonia: Molecular Phylogeny and Evolution of Fungi.

[10] Pak J, Tucci VT, Vincent AL, Sandin RL, Greene JN (2008). Mucormycosis in immunochallenged patients. J. Emerg. Trauma Shock..

[11] Petrikkos G (2012). Epidemiology and clinical manifestations of mucormycosis. Clin Infect Dis..

[12] Roden MM (2005). Epidemiology and outcome of zygomycosis: a review of 929 reported cases. Clin Infect Dis..

[13] Lackner M, Caramalho R, Lass-Flörl C (2014). Laboratory diagnosis of mucormycosis: current status and future perspectives. Future Microbiol..

[14] Herbrecht R (2002). Voriconazole versus amphotericin B for primary therapy of invasive aspergillosis. N Engl J Med..

[15] Siwek GT (2006). Incidence of invasive aspergillosis among allogeneic hematopoietic stem cell transplant patients receiving voriconazole prophylaxis. Diagn Microbiol Infect Dis..

[16] Cornely OA (2014). ESCMID and ECMM joint clinical guidelines for the diagnosis and management of mucormycosis 2013. Clin Microbiol Infect..

[17] Alastruey-Izquierdo A (2009). *In vitro* activity of antifungals against zygomycetes. Clin. Microbiol. Infect..

[18] Almyroudis NG, Sutton DA, Fothergill AW, Rinaldi MG, Kusne S (2007). *In vitro* susceptibilities of 217 clinical isolates of zygomycetes to conventional and new antifungal agents. Antimicrob. Agents Chemother..

[19] Pagano L (2013). Combined antifungal approach for the treatment of invasive mucormycosis in patients with hematological diseases: a report from the SEIFEM and FUNGISCOPE registries. Haematol..

[20] Ritz N (2005). Failure of voriconazole to cure disseminated zygomycosis in an immunocompromised child. Eur. J. Pediatr..

[21] Ustun C, Farrow S, DeRemer D, Fain H, Jillella AP (2007). Early fatal *Rhizopus* infection on voriconazole prophylaxis following allogeneic stem cell transplantation. Bone Marrow Transplant..

[22] Wooley DW (1944). Some new aspects of the relationships of chemical structure to biological activity. Science.

[23] Arendrup MC (2014). Update on antifungal resistance in *Aspergillus* and *Candida*. Clin Microbiol Infect..

[24] Garcia-Effron G, Kontoyiannis DP, Lewis RE, Perlin DS (2008). Caspofungin-resistant *Candida tropicalis* strains causing breakthrough fungemia in patients at high risk for hematologic malignancies. Antimicrob Agents Chemother..

[25] Gregson L (2013). *In vitro* susceptibility of *Aspergillus fumigatus* to isavuconazole: correlation with itraconazole, voriconazole, and posaconazole. Antimicrob Agents Chemother..

[26] Singh J, Rimek D, Kappe R (2005). *In vitro* susceptibility of 15 strains of zygomycetes to nine antifungal agents as determined by the NCCLS M38-A microdilution method. Mycoses.

[27] Nelson DR (2009). The Cytochrome P450 Homepage. Hum Genomics.

[28] Leonardelli F (2016). *Aspergillus fumigatus* intrinsic fluconazole resistance is due to the naturally occurring T301I substitution in Cyp51Ap. Antimicrob Agents Chemother..

[29] Sun QN, Fothergill AW, McCarthy DI, Rinaldi MG, Graybill JR (2002). *In vitro* activities of posaconazole, itraconazole, voriconazole, amphotericin B, and fluconazole against 37 clinical isolates of zygomycetes. Antimicrob. Agents Chemother..

[30] Chakrabarti A (2011). Drug resistance in fungi – an emerging problem. Regional Health Forum.

[31] Pongas GN, Lewis RE, Samonis G, Kontoyiannis DP (2009). Voriconazole-associated zygomycosis: a significant consequence of evolving antifungal prophylaxis and immunosuppression practices?. Clin Microbiol Infect..

[32] Caramalho R (2015). Etest cannot be recommended for *in vitro* susceptibility testing of mucorales. Antimicrob Agents Chemother..

[33] Syed K, Mashele SS (2014). Comparative analysis of P450 signature motifs EXXR and CXG in the large and diverse kingdom of fungi: identification of evolutionarily conserved amino acid patterns characteristic of P450 family. PLoS ONE.

[34] Chau AS, Chen G, McNicholas PM, Mann PA (2006). Molecular basis for enhanced activity of posaconazole against *Absidia corymbifera* and *Rhizopus oryzae*. Antimicrob Agents Chemother..

[35] Chau AS, Mendrick CA, Sabatelli FJ, Loebenberg D, McNicholas PM (2004). Application of real-time quantitative PCR to molecular analysis of *Candida albicans* strains exhibiting reduced susceptibility to azoles. Antimicrob Agents Chemother..

[36] Delye C, Laigret F, Corio-Costet MF (1997). A mutation in the 14 alpha-demethylase gene of *Uncinula necator* that correlates with resistance to a sterol biosynthesis inhibitor. Appl Environ Microbiol..

[37] Leroux P, Walker AS (2011). Multiple mechanisms account for resistance to sterol 14 alpha-demethylation inhibitors in field isolates of *Mycosphaerella graminicola*. Pest Manag Sci..

[38] Lescar J (2014). *Aspergillus fumigatus* harbouring the sole Y121F mutation shows decreased susceptibility to voriconazole but maintained susceptibility to itraconazole and posaconazole. J. Antimicrob Chemother..

[39] Morales IJ (2003). Characterization of a lanosterol 14 alpha-demethylase from *Pneumocystis carinii*. Am J Respir Cell Mol Biol..

[40] Sionov E (2012). Identification of a C*ryptococcus neoformans* cytochrome P450 lanosterol 14 alpha-demethylase (Erg11) residue critical for differential susceptibility between fluconazole/voriconazole and itraconazole/posaconazole. Antimicrob Agents Chemother..

[41] Vandeputte P (2005). Mechanisms of azole resistance in a clinical isolate of *Candida tropicalis*. Antimicrob Agents Chemother..

[42] Wheat LJ (2006). Activity of newer triazoles against *Histoplasma capsulatum* from patients with AIDS who failed fluconazole. J Antimicrob Chemother..

[43] Sagatova AA (2016). Triazole resistance mediated by mutations of a conserved active site tyrosine in fungal lanosterol 14alpha-demethylase. Sci Rep..

[44] Monk BC (2014). Architecture of a single membrane spanning cytochrome P450 suggests constraints that orient the catalytic domain relative to a bilayer. Proc Natl Acad Sci..

[45] Garcia-Effron G (2008). Rapid detection of triazole antifungal resistance in *Aspergillus fumigatus*. J Clin Microbiol..

[46] Sionov E, Chang YC, Garraffo HM, Kwon-Chung KJ (2009). Heteroresistance to fluconazole in *Cryptococcus neoformans* is intrinsic and associated with virulence. Antimicrob Agents Chemother..

[47] Edlind TD, Henry KW, Metera KA, Katiyar SK (2001). A*spergillus fumigatus* CYP51 sequence: potential basis for fluconazole resistance. Med Mycol..

[48] Dannaoui E, Meletiadis J, Mouton JW, Meis JF, Verweij PE (2003). *In vitro* susceptibilities of zygomycetes to conventional and new antifungals. J. Antimicrob. Chemother..

[49] Guinea J, Peláez T, Recio S, Torres-Narbona M, Bouza E (2008). *In vitro* antifungal activities of isavuconazole (BAL4815), voriconazole, and fluconazole against 1,007 Isolates of zygomycete, *Candida*, *Aspergillus*, *Fusarium*, and *Scedosporium* species. Antimicrob Agents Chemother..

[50] Sabatelli F (2006). *In vitro* activities of posaconazole, fluconazole, itraconazole, voriconazole, and amphotericin B against a large collection of clinically important molds and yeasts. Antimicrob Agents Chemother..

[51] Lamaris GA (2009). Increased virulence of zygomycetes organisms following exposure to voriconazole: a study involving fly and murine models of zygomycosis. J Infect Dis..

[52] Horn F (2015). Draft genome sequences of symbiotic and nonsymbiotic *Rhizopus microsporus* strains CBS 344.29 and ATCC 62417. Genome Announc..

[53] Nelson DR (1999). Cytochrome P450 and the individuality of species. Arch Biochem Biophys..

[54] Rex, J. H. *et al*. Reference method for broth dilution antifungal susceptibility testing of filamentous fungi; Approved Standard—second edition *Clinical and Laboratory Standards Institute*, *Wayne PA*. **28** (2008).

[55] Cuenca-Estrella M (2007). Multicentre determination of quality control strains and quality control ranges for antifungal susceptibility testing of yeasts and filamentous fungi using the methods of the Antifungal Susceptibility Testing Subcommittee of the European Committee on Antimicrobial Susceptibility Testing (AFST-EUCAST). Clin. Microbiol. Infect..

[56] Ma L-J (2009). Genomic analysis of the basal lineage fungus *Rhizopus oryzae* reveals a whole-genome duplication. PLoS Genet..

[57] Lee SC (2014). Analysis of a food-borne fungal pathogen outbreak: virulence and genome of a *Mucor circinelloides* isolate from yogurt. mBio.

[58] Wöstemeyer J (1985). Strain-dependent variation in ribosomal DNA arrangement in *Absidia glauca*. FEBS J..

[59] Rio DC, Ares M, Hannon GJ, Nilsen TW (2010). Purification of RNA using TRIzol (TRI reagent). Cold Spring Harb Protoc..

[60] Webb, B., Sali, A. Comparative protein structure modeling using MODELLER. In *Curr. Protoc. Bioinform*. **47**(unit 5.6), 1–32 (2014). 10.1002/0471250953.bi0506s47 25199792

[61] Notredame C, Higgins DG, Heringa J (2000). T-Coffee: A novel method for fast and accurate multiple sequence alignment. J Mol Biol..

