# Supplementary material for: Intrinsic short-tailed azole resistance in mucormycetes is due to an evolutionary conserved aminoacid substitution of the lanosterol 14α-demethylase
Source: Sci Rep. 2017 Nov 21;7:15898. doi: 10.1038/s41598-017-16123-9 (PMC5698289; doi:10.1038/s41598-017-16123-9)
Supplement: Supplementary file 1 — Supplementary Information [file 41598_2017_16123_MOESM1_ESM.pdf]

Supplementary Information

Intrinsic short-tailed azole resistance in mucormycetes is due to an evolutionary conserved aminoacid substitution of the lanosterol 14α-demethylase.

Rita Caramalho [1], Joel D. A. Tyndall [2], Brian C. Monk [3], Thomas Larentis [1], Cornelia Lass-Flörl [1] and Michaela Lackner [1*]

[1] Division of Hygiene and Medical Microbiology, Medical University of Innsbruck, Schöpfstraße, number 41, 2nd floor, A-6020 Innsbruck, Austria;

[2] School of Pharmacy, University of Otago, Dunedin 9054, New Zealand;

[3] Sir John Walsh Research Institute and the Department of Oral Sciences, New Zealand's National Centre for Dentistry, University of Otago, Dunedin 9054, New Zealand.

[*] corresponding author:

Ass. Prof. Priv.-Doz. Mag. Dr. rer. nat. Michaela Lackner Division of Hygiene and Medical Microbiology, Medical University of Innsbruck, Schöpfstraße 41, 6020 Innsbruck, Austria, phone: +43 512 9003 70725, Email: Michaela.Lackner@i-med.ac.at

Table S1. *In vitro* antifungal susceptibility of *Mucor circinelloides, Rhizopus microsporus*, and *Rhizopus arrhizus* against fluconazole and triadimenol.

	Median MIC (range) [a] (mg/L)	
**Species (n. strains tested)**	**FLC**	**TDM**
R. arrhizus (n=6)	>64.00 (>64.00)	>16.00 (>16.00)
R. microsporus (n=6)	>64.00 (>64.00)	>16.00 (>16.00)
M. circinelloides (n=6)	>64.00 (>64.00)	>16.00 (>16.00)

Table legend. [a] FLC and TDM susceptibility according to CLSI standard microbroth dilution method; MIC. minimum inhibitory concentration; FLC, fluconazole; TDM, triadimenol.

Table S2. Accession numbers corresponding to fungal LDMs aligned with the CYP51 F5 and CYP51 F1 consensus sequences of Mucorales species *Rhizopus arrhizus*, *Rhizopus microsporus*, and *Mucor circinelloides.*

**species**	**LDM Genbank accession number**
Saccharomyces cerevisae	NP_011871
Histoplasma capsulatum	AAU01158.1
Coccidioides posadasii	KMM65971.1
Coccidioides immitis	XP_001246802.1
Pneumocystis carinii	AAO38776.1
Scedosporium apiospermum	KEZ44212.1
Zymoseptoria tritici	AKA94187.1
Uncinula necator	O14442*
Aspergillus fumigatus	ACF18581.1
Aspergillus flavus	XP_002375123
Candida albicans	ADO21804.1
Candida glabrata	XP_445876.1
Candida tropicalis	AMR44154.1
Candida dubliniensis	AAK57519.1
Malassezia globosa	XP_001730619.1
Cryptococcus neoformans var. grubii	AEX20237.1
Cryptococcus gattii	AEQ63274.1
Mucor ambiguus CYP51 F5/CYP51 F1	A0A0C9M5D0*/A0A0C9M5R4*
Parasitella parasitica CYP51 F5/CYP51 F1	A0A0B7NND2*/ A0A0B7NSN9*
Absidia glauca CYP51 F5/CYP51 F1	ABSGL_12517.1 scaffold 12955*/ABSGL_12606.1 scaffold 13066*

Table legend. LDM. lanosterol 14-α demethylase; * accession number retrieved from UniProt database (http://www.uniprot.org/uniprot).

Table S3. Conditions for the q-PCRs developed for CYP51 F5, CYP51 F1.

qPCR gene target	species	direction	primer sequence (5´to 3´)	amplicon size (bp)	targeted triplet	spanned CDS region	translated aa	LDM location
CYP51 F5	*R. arrhizus*	For	GGACCCGACGGKAACCAATT	208	TTY	positions 385 to 387	phenylalanine	129
		Rev	ATTCCTCGATRATCAGCGGC					
	R. microsporus	For	AAGCGCGTCACTGCATGCCT	196				
		Rev	ACGACTCGATATTTAAYCCGCT					
	M. circinelloides	For	CTCTTGGGTYCCCATCATGG	202				
		Rev	GTTGTAAGCAGCAGCAGCAG					
CYP5 F1	*R. arrhizus*	For	TGAATGCAGACGGAAACCAA	247	TAY	positions 379 to 381	tyrosine	127
		Rev	CCCGTAGGCTTCTTGTAGTTTT					
	R. microsporus	For	ATATGGTTGGCCGACGTGTT	223				
		Rev	GGAACGTGTTGACGGAATGC					
	M. circinelloides	For	CCGTTCTCTTGGGTGCTGAT	203		positions 388 to 390		130
		Rev	GGGCACATGTTGACGRAARC					
Actin	all 3 species*	For	TCCTGGTATTGCYGAYCGT	128	N.A			
		Rev	GAGARGCYAARATGGAACCA					

Table legend. The primers, amplicon sizes, targeted triplets, the regions where these are located and the position of each respective translated amino acid used to confirm the expression of both CYP51 genes are presented. The qPCR primers and amplicon sizes used for the actin as normalization gene are also depicted. qPCR, quantitative reverse transcription PCR; for, forward; rev, reverse; bp, base pairs; CDS, coding sequence; aa, amino acid; LDM. lanosterol 14-α demethylase; N.A. not applicable; **M. circinelloides, R. microsporus*, and *R. arrhizus*.

Table S4. Oligonucleotide primers used for amplification and sequencing of the pairs of paralogous CYP51 genes in three Mucorales species.

primer name	species	locus[a]	direction	sequence (5' to 3')	AT (°C)	amplicon size (bp)
**Muci1_Fw**	*M. circinelloides*	*CYP51*A F1	**For**	**ACC TCA CTC CCC TCT CTC TCC A**	58	1800
Muci1_R1			Rev	CGG TGA CCT TCT TGC CGA CCA		
Muci1_F2			For	TCG AGT TGG CCG CTC GTC CT		
**Muci1_c2**			**Rv**	**CAG GCA CAA CAA CCA TAG AAG TGT**		
**Muci2_Fw**		*CYP51*B F5	**For**	**ACC GAT GGT GAC ACG CCG TA**	60	1967
Muci2_F1.1			For	TGG GCC CTG ATG GCA ACC AA		
Muci2_F2.1			For	TCT GCT CTC CCT GAC CAC CA		
Muci2_R1			Rev	TGT AGG AAT CGA CGA CAC CAG TCT		
**Muci2_Rv**			**Rev**	**GCG CAC ATG TAT TTG TCC AGT AGC**		
**Rmicr_CYP51A_FW**	*R. microsporus*	*CYP51*A F1	**For**	CAG ACC CGT CCG TTA CAC AA	56	1779
Rmicr_CYP51A_F2			For	AAC GCA AAG CAG AAT TTG GC		
Rmicr_CYP51A_R1			Rev	AGA CAA CTT CAG GGC CAA ACA		
Rmicr_CYP51A_F3			For	GGA ACA CCC TTA CCT GAT CAT CA		
Rmicr_CYP51A_F4			For	GCG TGC CTA AGC CTG ACT AT		
Rmicr_CYP51A_R2			Rev	CAG AGG TGT GTT GAC CAC CA		
**Rmicr_CYP51A_RV**			**Rev**	**TGC TCC GTC ACT ACA CAT GT**		
**Rmic_Fw**		*CYP51B* F5	**For**	**TGC CGG GCT CGA TGA CCG AT**	60	1568
Rmic_F1			For	AAGCGCGTCACTGCATGCCT		
Rmic_R1			Rev	TCT GCA ACG ACA CGG CGC AT		
Rmic_F2			For	TGG TCC AGA GGT TCG TAA CGC		
**Rmic_Rv**			**Rev**	**CGG TGG CGT CCA GCA CCA AA**		
**Rdel_Cyp51A_17757fw**	*R. arrhizus*	*CYP51A* F5	**For**	**GTT CGA TCT GCT ACA AAC GCC**	58	2032

Rdel2.F2		For	AAA CGC GTC ACG GCC TGC TT		
Rdel2.R1		Rev	CCA GGC GGC AGT GGT GGA AG		
Rdel2.F3		For	TAC GG CCT CCC GTA CCC TGC		
Rdel2.Rv		Rev	ACA GGG TTC CAT CCG TTC CAG T		
**Rdel_CYP51A_19851v**		**Rev**	**TTG ATG CTG ATG GCG CTC TAT**		
**Rdel_F1**	*CYP51B* F1	**For**	**TGC AAA TGG AGC CGC GAC GA**	58	1932
Rdel_R1		Rev	TCG GAT GAG TTG TGT TAT CGC TTC		
Rdel_F1.1		For	TGC TCC TCA CAG CGT ATT CAT GG		
Rdel_F2.1		For	CGT ACC TGA CCA TCA CAT TGC CG		
**Rdel_Rv**		**Rev**	**ACG TCA CAT AGT TAA ACA GCC A**		

Table legend: [a] locus names *CYP51A* (F1) and *CYP51B* (F5) were attributed according to Nelson´s database, as a result of BLAST analysis against P450 protein families. For, forward; Rev, reverse; AT, annealing temperature °C; bp. base pairs; primers shown in bold were used for both DNA amplification and Sanger sequencing.

Figure S1.

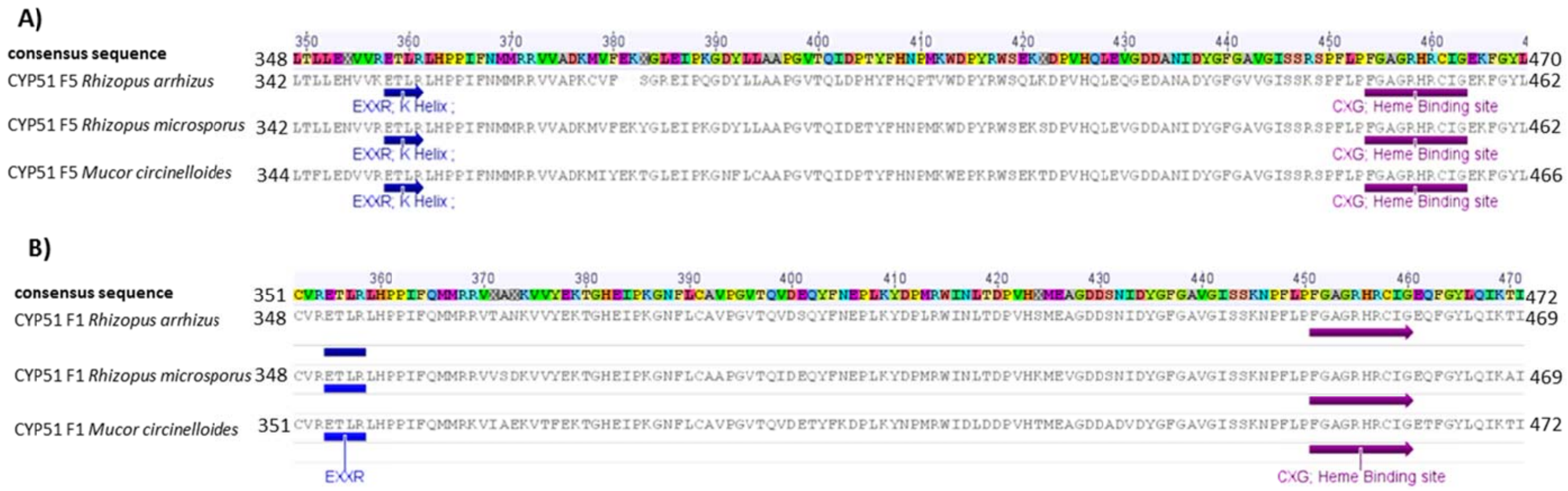

Figure legend. Sequence alignment of the two orthologous paralogous CYP51 genes showing consensus sequences retrieved for the species *R. arrhizus*, *R. microspores* and *M. circinelloides* and the location of the conserved motifs EXXR (ETLR in both protein sequences) marked in blue, and CXG (CIG in both protein sequences) marked in purple.

Figure S2.

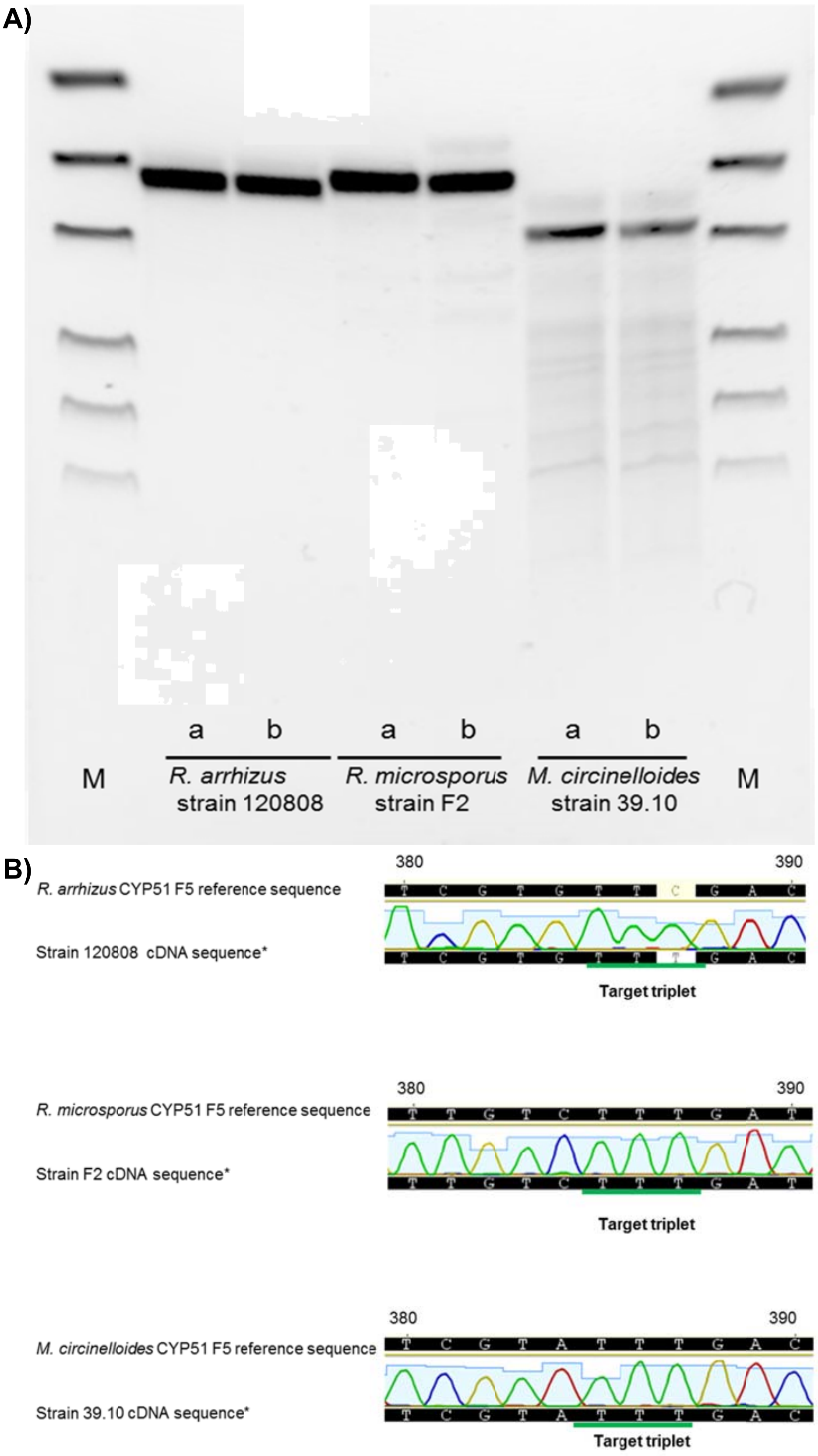
A)
a b a b a b
M
R. arrhizus strain 120808
R. microsporus strain F2
M. circinelloides strain 39.10
M
B)
380
390
R. arrhizus CYP51 F5 reference sequence
Strain 120808 cDNA sequence*
Target triplet
380
390
R. microsporus CYP51 F5 reference sequence
Strain F2 cDNA sequence*
Target triplet
380
390
M. circinelloides CYP51 F5 reference sequence
Strain 39.10 cDNA sequence*
Target triplet

Figure legend. A) A 2% w/v agarose gel depicting cDNA products from three isolates of the species *R. arrhizus*, *R. microsporus,* and *M. circinelloides*, (CBS strain 120808, clinical isolate F2 and clinical isolate 39.10, respectively) obtained from the qPCR performed for expression of *CYP51* F5; **a**, cDNA product obtained after 48 h growth without exposure to the ITC, and **b** cDNA product obtained after exposure for 48h to 0.50 mg/L of ITC; M, 100 bp marker. **B)** Sequence analysis of each cDNA product shown in **A)** after ITC exposure. The sequence obtained using just the forward primer is shown (both primers were used for sequence analysis and assembly). The target triplet (positions 385 to 387) in the *CYP51* F5 CDS of the three species is underlined in green. For a better visualization the image was color-invented.

Figure S3. *Rhizopus arrhizus* and *R. microsporus* strains exhibit variable *in vitro* minimum inhibitory concentrations for voriconazole according to CLSI microbroth dilution method.

Phenotype VCZ MIC[1] (mg/L)	Species/strain number[2]	Lanosterol 14-α demethylase F5 partial sequence, mapped to reference (130–150)
n.a.	***Saccharomyces cerevisae* ERG11[3]**	T T P V F G K G V I **Y** D C P N S R L M E Q
4.00	*R. arrhizus* / 120808 LDM F5	T K Y V F G N D I V **F** D T A H S I F M E Q
16.00	*R. arrhizus* / 136236 LDM F5	T K Y V F G N D I V **F** D T A H S V F M E Q
>16.00	*R. arrhizus* / 136239 LDM F5	T K Y V F G N D I V **F** D T A H S V F M E Q
4.00	*R. microsporus* / 71.11 LDM F5	T K Y V F G N D V V **F** D T A H S I F M E Q
8.00	*R. microsporus* / AS 40 LDM F5	T K Y V F G N D V V **F** D T A H S I F M E Q
16.00	*R. microsporus* / 30.10 LDM F5	T K Y V F G N D V V **F** D T A H S I F M E Q

Figure legend. Voriconazole (VCZ) minimum inhibitory concentrations (MIC[1]) were determined according to CLSI standard microbroth dilution method [54] ; [2] strain information is given in a previous published study [32]; the partial LDM F5 sequence with amino acid substitution Y140F (*S. cerevisae* numbering) is mapped to the reference *S. cerevisiae* LDM [3] (accession number given in Supplementary Table S2): LDM F5, lanosterol 14-α demethylase primary sequence translated from CYP51 F5 gene and named according to Nelson's P450 Database [27]; location of the amino acid substitution in all Mucorales strains highlighted in bold; n.a. not applicable.
